# Supplementary material for: Surface Microbiome Profiling of Dental Elevators Using Third‐Generation Sequencing: Implications for Infection Control in Dental Practice
Source: Microbiologyopen. 2025 Dec 8;14(6):e70178. doi: 10.1002/mbo3.70178 (PMC12685768; doi:10.1002/mbo3.70178)
Supplement: Supplementary file 1 — customer_backup. [file MBO3-14-e70178-s001.zip › customer_backup/customer_backup/taxa_summary/heatmap/treat/treat.family.reabundance.heatmap.pdf]

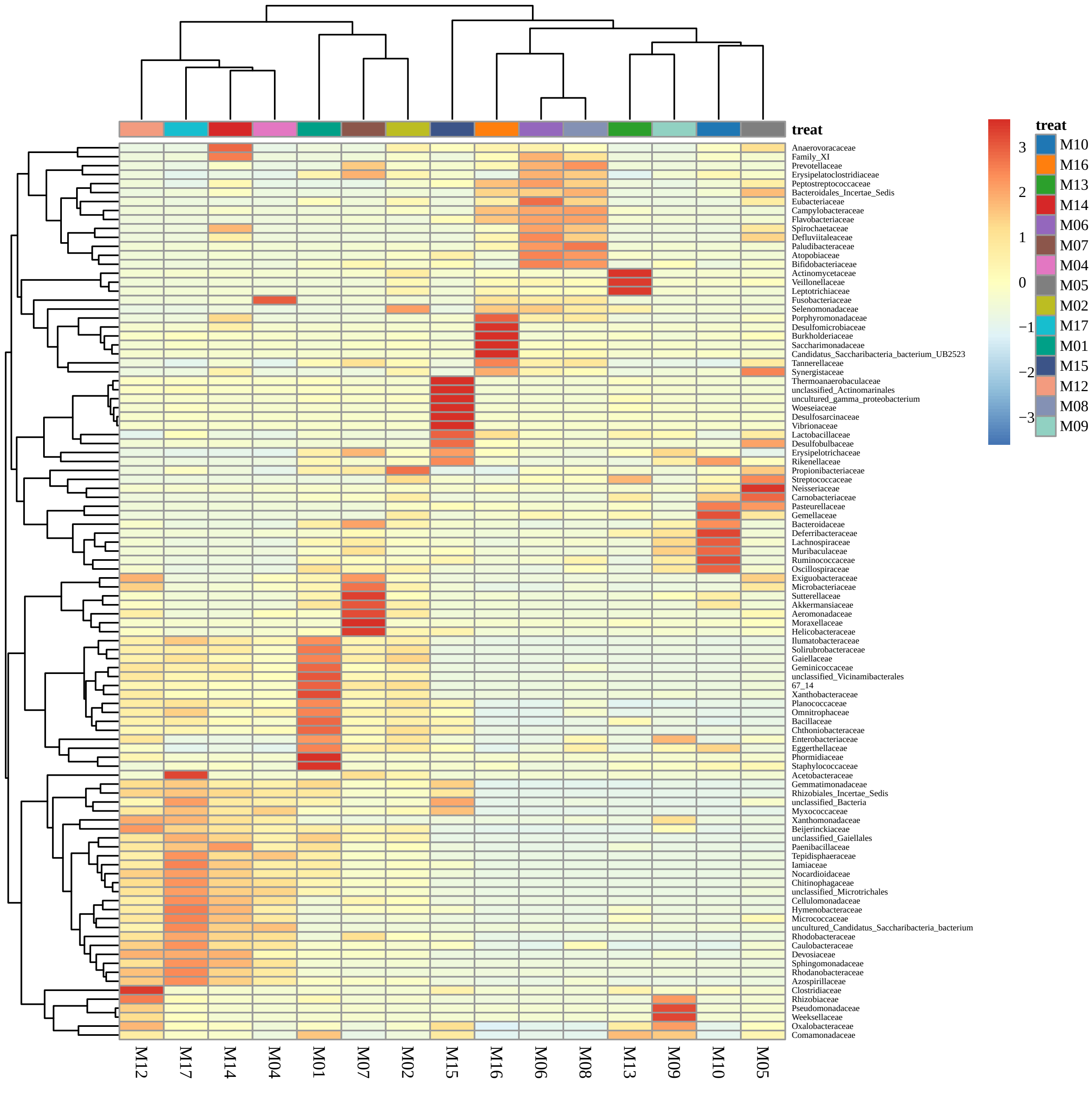
treat
treat
M10
M16
M13
M14
M06
M07
M04
M05
M02
M17
M01
M15
M12
M08
M09
3
2
1
0
−1
−2
−3
Anaerovoracaceae
Family_XI
Prevotellaceae
Erysipelatoclostridiaceae
Peptostreptococcaceae
Bacteroidales_Incertae_Sedis
Eubacteriaceae
Campylobacteraceae
Flavobacteriaceae
Spirochaetaceae
Defluviitaleaceae
Paludibacteraceae
Atopobiaceae
Bifidobacteriaceae
Actinomycetaceae
Veillonellaceae
Leptotrichiaceae
Fusobacteriaceae
Selenomonadaceae
Porphyromonadaceae
Desulfomicrobiaceae
Burkholderiaceae
Saccharimonadaceae
Candidatus_Saccharibacteria_bacterium_UB2523
Tannerellaceae
Synergistaceae
Thermoanaerobaculaceae
unclassified_Actinomarinales
uncultured_gamma_proteobacterium
Woeseiaceae
Desulfosarcinaceae
Vibrionaceae
Lactobacillaceae
Desulfobulbaceae
Erysipelotrichaceae
Rikenellaceae
Propionibacteriaceae
Streptococcaceae
Neisseriaceae
Carnobacteriaceae
Pasteurellaceae
Gemellaceae
Bacteroidaceae
Deferribacteraceae
Lachnospiraceae
Muribaculaceae
Ruminococcaceae
Oscillospiraceae
Exiguobacteraceae
Microbacteriaceae
Sutterellaceae
Akkermansiaceae
Aeromonadaceae
Moraxellaceae
Helicobacteraceae
Ilumatobacteraceae
Solirubrobacteraceae
Gaiellaceae
Geminicoccaceae
unclassified_Vicinamibacterales
67_14
Xanthobacteraceae
Planococcaceae
Omnitrophaceae
Bacillaceae
Chthoniobacteraceae
Enterobacteriaceae
Eggerthellaceae
Phormidiaceae
Staphylococcaceae
Acetobacteraceae
Gemmatimonadaceae
Rhizobiales_Incertae_Sedis
unclassified_Bacteria
Myxococcaceae
Xanthomonadaceae
Beijerinckiaceae
unclassified_Gaiellales
Paenibacillaceae
Tepidisphaeraceae
Iamiaceae
Nocardioidaceae
Chitinophagaceae
unclassified_Microtrichales
Cellulomonadaceae
Hymenobacteraceae
Micrococcaceae
uncultured_Candidatus_Saccharibacteria_bacterium
Rhodobacteraceae
Caulobacteraceae
Devosiaceae
Sphingomonadaceae
Rhodanobacteraceae
Azospirillaceae
Clostridiaceae
Rhizobiaceae
Pseudomonadaceae
Weeksellaceae
Oxalobacteraceae
Comamonadaceae
M12
M17
M14
M04
M01
M07
M02
M15
M16
M06
M08
M13
M09
M10
M05
